# Development and Characterization of Titanium Dioxide Ceramic Substrates with High Dielectric Permittivities

**DOI:** 10.3390/ma13020386

**Published:** 2020-01-14

**Authors:** Antonio E. Freitas, Taise M. Manhabosco, Ronaldo J. C. Batista, Alan K. Rêgo Segundo, Humberto X. Araújo, Fernando Gabriel S. Araújo, Adilson R. Costa

**Affiliations:** 1 REDEMAT-Laboratório de Engenharia de Superfícies e Técnicas Afins–LESTA, Centro Histórico, Universidade Federal de Ouro Preto, 35400-000 Ouro Preto, Brazil; adilson@ufop.edu.br; 2 Departamento de Tecnologia em Engenharia Civil, Computação e Humanidades–DTECH, Campus Alto Paraopeba, Universidade Federal de São João del Rei, 36301-158 Ouro Branco, Brazil; 3 Departamento de Física–DEFIS/REDEMAT, Campus Universitário Morro do Cruzeiro, Universidade Federal de Ouro Preto, 35400-000 Ouro Preto, Brazil; taisemanhabosco@gmail.com (T.M.M.); batista.rjc@gmail.com (R.J.C.B.); fgabriel@ufop.edu.br (F.G.S.A.); 4 Departamento de Engenharia de Controle e Automação–DECAT, Campus Universitário Morro do Cruzeiro, Universidade Federal de Ouro Preto, 35400-000 Ouro Preto, Brazil; alankardek@ufop.edu.br; 5 Colegiado de Engenharia Elétrica, Universidade Federal do Tocantins, 77600-000 Palmas, Brazil; hxaraujo@uft.edu.br

**Keywords:** titanium dioxide, ceramic substrate, permittivity, solid state sintering, porosity

## Abstract

Titanium dioxide substrates have been synthesized by means of solid-state reactions with sintering temperatures varying from 1150 °C up to 1350 °C. X-ray diffraction and scanning electron microscopy (SEM) where employed to investigate the crystal structure, grain size and porosity of the resulting samples. The obtained ceramics are tetragonal (rutile phase) with average grain sizes varying from 2.94 µm up to 5.81 µm. The average grain size of samples increases with increasing temperature, while the porosity decreases. The effect of microstructure on the dielectric properties has been also studied. The reduction of porosity of samples significantly improves the dielectric parameters (relative dielectric permittivity and loss tangent) in comparison to those of commercial substrates, indicating that the obtained ceramic substrates could be useful in the miniaturization of telecommunication devices.

## 1. Introduction

Dielectric materials were initially used in telecommunication as base materials for the fabrication of radio frequency and microwave components such as tunable filters, amplifiers and oscillators [1,2,3,4]. Later, these materials were used as dielectric resonators for radio waves in antennas, opening up an important field in telecommunications [5]. Among different dielectric materials, ceramics present excellent properties like high resistance to environmental degradation, resistance to oxidation and appropriate hardness. In addition, ceramics present suitable electromagnetic properties, like high relative dielectric permittivity (*ε*_r_) and magnetic permeability (μ) [5], which favor their use in telecommunication devices. In fact, the dimension of an antenna is of the order of λ_0_*ε*^−0.5^, where λ_0_ is the free-space wavelength and *ε* is the dielectric constant, therefore the antenna size can significantly reduce with by choosing a substrate with a high value of *ε*_r_ [6,7].

Among different antenna designs, the microstrip antenna is one that can be constructed using ceramic substrates. Essentially, such an antenna is composed of a dielectric substrate between a thin metal patch, which works as the electromagnetic signal radiator, and a thin metal ground plane [8,9,10,11,12]. Microstrip antennas provide suitable performance, however their limited bandwidth, low gain and low power have limited their use [13,14,15]. In order to overcome those problems, new ceramic substrates like bismuth niobate [16], niobium pentoxide [17], zirconia titanate [18], magnesium silicate [19] and titanium dioxide [20,21], have been used. Such ceramics have relatively high dielectric permittivity and low dielectric loss (loss tangent), which allows size reduction and bandwidth increases.

In the present study, we investigated titanium dioxide as a ceramic dielectric substrate for potential applications in telecommunication devices, in particular microstrip antennas. Our choice was based on the low price of titanium dioxide and its inertness to the human body. The substrates were fabricated by means of a solid-state reaction. Such a technique produces quality ceramics at low cost, which favors the economical viability for the antennas and other ceramic-based electronic devices. Because the dielectric properties of the substrates play a fundamental role in the performance and size of antennas and other electronic devices, we evaluated the effects of substrates microstructure on their dielectric permittivity and dielectric losses.

## 2. Materials and Methods

TiO_2_ substrates were fabricated from TiO_2_ powder (99.7% purity, AlfaAesar, (Ward Hill, MA, USA). Preliminary studies indicated the necessity of using a binder to ensure compaction and avoid delamination. The binder used was 6.5 wt % polyvinyl alcohol (PVA) diluted in water (80 g/Kg). The TiO_2_ powder and PVA binder were manually mixed during half an hour and ball milled for further two hours. The resulting powder was uniaxially cold compacted under pressure of 138 MPa in the form of green pellets with a diameter of 30 mm and thickness about 1.6 mm. The green pellets were finally sintered in air atmosphere at different temperatures in order to evaluate the microstructure influence on electromagnetic parameters. The sintering was performed at 1150, 1200, 1250 and 1300 °C for 4 h and at 1230, 1300 and 1350 °C for 12 h. Samples will be referred in the text as 1150/4, 1200/4, 1250/4, 1300/4, 1230/12, 1300/12 and 1350/12, respectively. A heating rate of 5 °C/min was applied to all sintering processes. The binder was removed at 450 °C during one hour. The microstructure of polished samples, revealed by thermal attack at 1100 °C for 30 min, was evaluated by Scanning electron microscopy (SEM). The analysis was performed in a JSM-6360LV instrument (JEOL Inc., Peabody, MA, USA) at an acceleration voltage of 30 kV. A gold coating was used to ensure ceramic conductivity. Polished samples were prepared according to the following procedure: samples were ground with SiC emery paper to 2000 grit and polished with 3 µm diamond paste. The crystal structure of ceramics sintered at different temperature and time was investigated by X-ray diffraction (XRD) patterns. The XRD patterns were obtained using a Bruker diffractometer (BRUKER AXS, Inc., Madison, WI, USA), model D2 Phaser, with Cu Kα radiation (λ = 1.5406 Å) with a scan range of 2*θ*, a step size of 0.02° and a measuring time of 0.500 s per point. Diffraction patterns were defined by comparing them with the crystallographic Joint Committee on Powder Diffraction Standards (JCPDS). The dielectric constant and dielectric loss of the samples were measured in a range of low frequencies (0.1–20 MHz) and at high frequencies at the resonant frequency (1.29 GHz and 2.0 GHz) by using the Hakki-Coleman method [22]. Measurements were performed at room temperature using an impedance analyzer Hioki IM7581-01 (Interworld Highway LLC, Long Branch, NJ, USA), for low frequencies, and a model N5230A vector network analyzer (VNA) from Agilent Technologies (Santa Clara, CA, USA) for resonant frequencies. In order to compare our results with that provided by the manufacturer, measurements were also performed for the commercial substrate FR4 (glass-reinforced epoxy laminate material). Results were quite similar. ImageJ open access software (Version 1.x, LOCI, University of Wisconsin, Madison, WI, USA) was applied to evaluate grain and pore sizes. Statistical analysis was performed using the Octave open access software (Version 5, John W. Eaton, Madison, WI, USA).

## 3. Results

**Figure 1:**
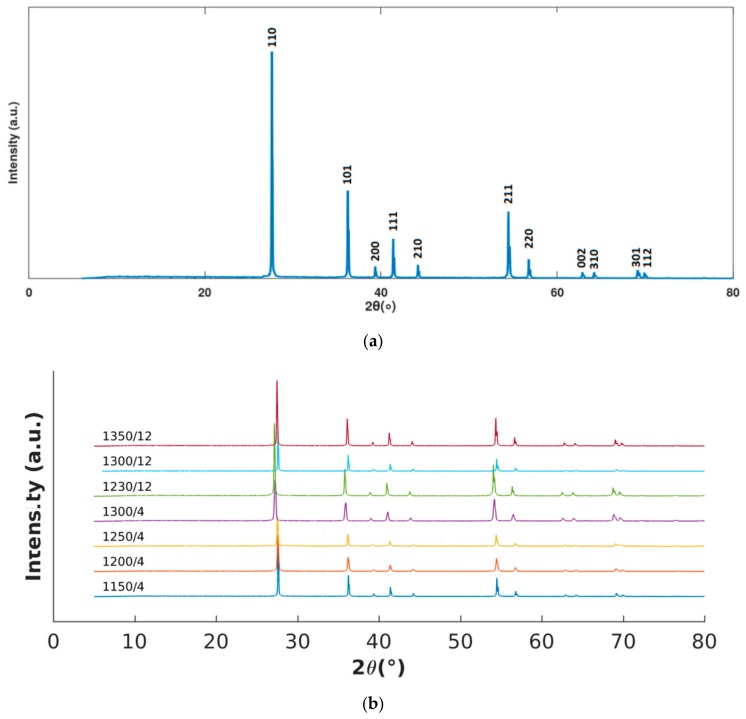
Pattern: (**a**) TiO_2_ samples sintered at 1300 °C for 12 h (1300/12). (**b**) XRD of all samples.

XRD measuments were performed for all samples sintered at different temperatures and times, which are presented in Figure 1. The XRD pattern of all samples is a single phase indexed as rutile (tetragonal system) according to JCPDS N° 21-1276. No amorphous phases related to the presence of PVA is identified. Other information obtained from XRD patterns is the crystallite size calculated using the Scherrer equation [23]:

(1) $$B\;\left(2\theta \right)=\frac{K\lambda }{\mathrm{Lcos}\theta }$$

where B(2*θ*) is the peak width at half the maximum intensity at a particular value of 2*θ*, K is a constant which depends on the crystallite shape but is generally taken as being about 1.0 for spherical particles, λ is the X-ray wavelength and L is the crystallite size.

The crystallite size was calculated from the most intense XRD peak (110) and is presented in Table 1. Where it is observed that the increase of temperature and time increases the mean size of the crystallite.

**Figure 2:**
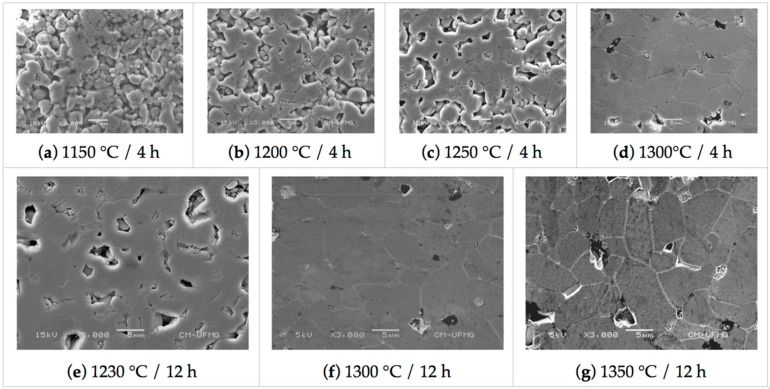
Images of TiO_2_ polished samples at different sintering temperatures and times.

Polished samples morphology of different sintering temperatures and time are presented in Figure 2. Samples sintered at 1150 °C, for four hours, are in the first sintering stage. Further stages are reach by increasing the sintering temperature and time, where the porosity decreases with increasing grain growth.

**Figure 3:**
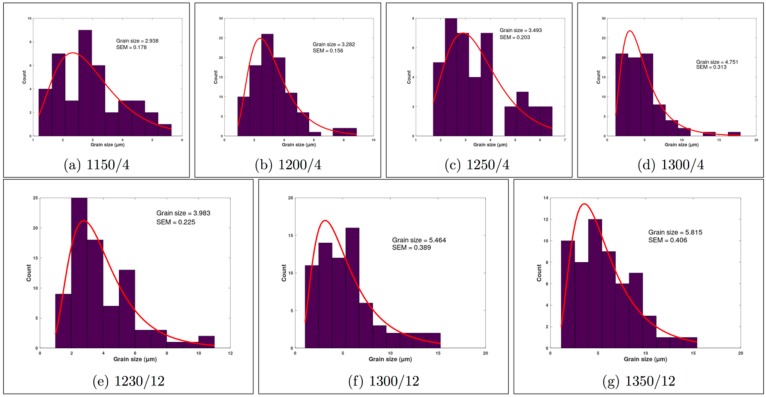
Size histograms at different sintering temperatures and times.

SEM images were computionally treated by means of the ImageJ and Octave softwares. The programs allowed calculating the distributions of mean grain size and pore size. Grain size histrograms are presented in Figure 3, while Table 2 presents the mean grain and pore size, with their standard error of the mean (SEM). It is observed for all samples that increasing temperature promote grain growth so as observed for crystallite size. Crystallite size is in the order of nm while grain size is about µm because a grain is composed of several crystallites.

It can be observed from Figure 3 and Table 2 that the pore size of samples sintered for 4 and 12 h tends to decrease in the way the temperature increases.

The densities were obtained from the ratio between mass and the volume of the pellets, whose dimensions are known [24]. Because density is an additive property and there are only two components in the sample (bulk TiO_2_ and pores), the total porosity percentage can be inferred from relative density. Their mean values are presented in the Table 3. As expected, the density increases with sintering temperature and time reaching a densification of approximately 86% meanwhile the porosity decreases up to 14%. According to the work of Kuang et. al. [25] the increase of sintering temperature of BaTiO_3_ samples from 1100 to 1250 °C can promote an increase in density up to values near the theoretical density, which in principle could also be applied to improve our ceramic densities. However, the disadvantages of using a high sintering temperature may include high production cost. Alternatively, as reported by Han et al. [26], an increase in green body densities could be a more efficient way to improve final density up to 98% of its theoretical value. In fact, the increase in density due sinterization processes, about 20%, obtained by Han et al. [26], is essentially the same we have obtained, therefore, the differences in samples final densities must be ascribed to differences in the green bodies’ initial densities. Then, the use more efficient compactation process, like isostatic pressing, or modifications of the powder should result in high-density green bodies and, therefore, high density samples.

**Figure 4:**
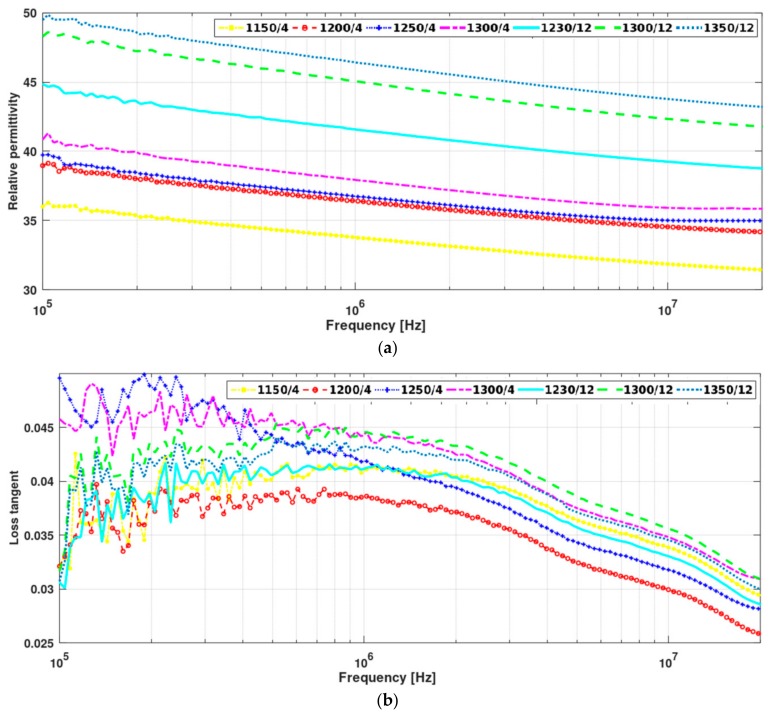
(**a**) Substrates relative dielectric permittivity and (**b**) loss tangent for TiO_2_ samples sintered at different temperatures and times.

Substrates relative dielectric permittivity and loss tangent, at low frequencies, were evaluate by means of an impedance meter equipment. The impedance meter imposes an electromagnetic wave, at a given frequency, and analyzes the reflected wave to obtain the dielectric parameters [27,28]. Dielectric permittivity and loss tangent were measured at high frequencies for the most promising sample to be applied on microstrip antennas. Measurements were performed by the Hakki-Coleman method at resonant frequencies (1.29 GHz and 2 GHz) [22]. Figure 4 presents the measured relative dielectric permittivity and loss tangent for low frequencies. Values at determined frequencies, 10^5^ Hz, 10^6^ Hz, 10^7^ Hz, 1.29 GHz and 2 GHz are reported in Table 4.

The values of electric permmittivity of the sintered samples shown in Table 4 are higher than those of commercial substrates, typically 9.6–10.1 in case of Al_2_O_3_ ceramic substrates for example [29]. The loss tangent of different TiO_2_ substrates presented a near zero value, as expected [30]. It can be observed that the electric permmittivity decreases when the frequency increases from 10^5^ to 10^7^ Hz. Such a behavior is due to relaxation effects associated with induced molecular dipoles, dielectric relaxation, and can be qualitatively described by classic Debye relaxation. At higher frequencies, resonance effects arising from the rotations or vibrations of atoms, ions, or electrons led the electric permitivitty to reach a maximum at 1.29 and 2 GHz. Among the produced substrates, the one with better properties to be used in telecommunication is the one sintered for 1350 °C during 12 h.

## 4. Discussion

As it can be seen in Table 1 and 2, the crystallite and grain size of rutile phase TiO_2_ substrates increases with increasing temperature and time. The changes in grain size with temperature/time are accompanied by a decrease in porosity, as it can be seen in Table 3. Those changes in the microstructure are consistent with the observed densification of samples, see Table 2. Densities between 0.6 and 0.65 indicate that samples sintered at 1150 °C are at the first stage of sintering [25,31], which is also characterized by the rounding necks shown in panel (a) of Figure 2. Further sintering stages, in which pores tends to disappear and grain growth occurs (see Figure 2), are achieved in as much as the sintering time and temperature increases. In fact, insights on the grain and pores kinetics can be obtained from the changes of the porous size and shape with temperature at two different sintering time (4 h and 12 h). The average pore size is expected to decrease, meanwhile, the average grain size is expected to increase with increasing temperature because of two combined effects: (i) atom mobility is thermal activated; and (ii) the driving force operative during sintering is the reduction of the excess energy associated with interfaces. Table 1 shows that grain sizes increase monotonically with temperature with larger grains being obtained for longer times as expected. Porosity also decreases monotonically with temperature and time, as it can be seen in Table 3.

The microstructure changes mentioned above have a significant effect on the dielectric constant at room temperature. We observed significantly increases in dielectric permittivity with loss tangent near zero, which may lead to an expressive size reduction of electronic devices in regards to the existing commercial ones, as alumina (*ε*_r_ = 9.8), gallium arsenide (*ε*_r_ = 12.9), Cordelite™ (a trademark of Trans Tech Company, Chandler, AZ, USA) (*ε*_r_ = 4.5) [30]. Previous studies on Ba_0.8_Sr_0.2_TiO_3_ and PbTiO_3_ ceramics [32,33] ascribe the changes in dielectric properties to the presence of grain boundaries and pores that constrains the electromagnetic waves.

**Figure 5:**
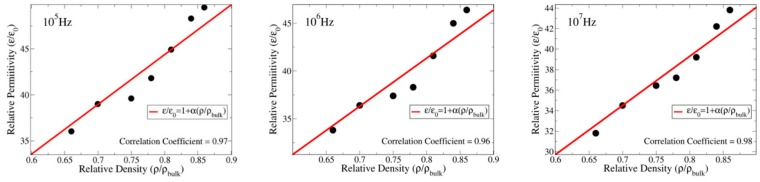
Dielectric permittivity as a function of the relative density measured at 10^5^, 10^6^ and 10^7^ Hz. The obtained correlation coefficients, 0.97, 0.96 and 0.98 indicate a strong correlation between density and permittivity. The linear behavior can be understood if one considers the sample permittivity as the sum of porous and bulk permittivity’s. The angular coefficient is given by: $\alpha =\frac{\varepsilon_{\mathrm{bulk}}-\varepsilon_{0}}{\varepsilon_{0}}$

In this work, we found that the dielectric permittivity of the samples is linearly proportional to their porosities, which are expressed in terms of the density of the sample relative to that of the perfect material, see Figure 5. Such a linear dependence can be explained by a simple analitic model in which the sample permittivity (*ε*) is due to the contributions of the pores and bulk, that is:

(2) $$\varepsilon =\frac{V_{p}\varepsilon_{p}+V_{\mathrm{bulk}}\varepsilon_{\mathrm{bulk}}}{V}=\frac{(V-V_{\mathrm{bulk}})\varepsilon_{p}+V_{\mathrm{bulk}}\varepsilon_{\mathrm{bulk}}}{V}=\varepsilon_{p}+(\varepsilon_{\mathrm{bulk}}-\varepsilon_{p})\frac{V_{\mathrm{bulk}}}{V}$$

where, *ε*_p_ and *ε*_bulk_ are the permittivities of pores and bulk, respectively. V_p_ and V are the total volume of the porous and the volume of the sample, and V_bulk_ = V − V_p_. Using V_bulk_/V = ρ/ρ_bulk_ and *ε*_p_ ≅ *ε*_0_, the above equation can be rewritten as follows:

(3) $$\frac{\varepsilon }{\varepsilon_{0}}=1+\frac{\left(\varepsilon_{\mathrm{bulk}}-\varepsilon_{0}\right)}{\varepsilon_{0}}\frac{\rho }{\rho_{\mathrm{bulk}}}$$

Note that such a model, which has a single adjustable parameter, is capable of fitting the experimental dada with a correlation coefficients equal to 0.96, 0.97 and 0.98. In addtion, the model provides a way to estimate the dielectric contants of the materials without pores (*ε*_bulk_/*ε*_0_) from samples that contains pores. In the case of TiO_2_, we estimate (*ε*_bulk_/*ε*_0_) = 49, 51 and 54 for 10^7^, 10^6^ and 10^5^ Hz, respectively, which can be seen as an upper bound for the relative permittivity of TiO_2_.

The effects of grain boundaries on the relative permittivity should be smaller than the effects of porosity because a model that takes into account effects of porosity, but not effects of grain boundaries, describes well the observed changes in permittivity.

## 5. Conclusions

Considering all tested conditions, we observed that the sintering of TiO_2_ at temperatures increasing from 1150 °C up to 1350 °C results in a significant increase in the relative dielectric permittivity values, which may allow their use as base material for the production of miniaturized telecommunication devices at low cost. Our experiments allow us to ascribe the observed increases in relative permittivities mainly to the reduction of porosity, with grain boundaries playing a minor role. Such a conclusion is corroborated by a simple analytic model that only takes into account the effects of porosity and is capable of fitting the experimental results.

## Figures and Tables

**Table 1:** Calculated crystalitte size of different sintered samples.

Sintering Condition (Temperature/Time)	1150/4	1200/4	1250/4	1300/4	1230/12	1300/12	1350/12
Crystallite size (nm)	48.0	49.6	58	59.5	62.7	68.8	69.0

**Table 2:** Size, pore size and total porosity of samples sintered at different temperatures and times.

Sample Surface	1150/4	1200/4	1250/4	1300/4	1230/12	1300/12	1350/12
Grain Size (µm)/ SEM	2.94/0.18	3.28/0.16	3.49/0.20	4.75/0.31	3.98/0.23	5.46/0.39	5.81/0.41
Pore Size (µm)/ SEM	3.16/0.21	2.71/0.21	2.02/0.16	1.68/0.23	1.53/0.16	1.46/0.15	1.87/0.26

**Table 3:** Substrate densities for different processing times and temperatures.

Sample	1150/4	1200/4	1250/4	1300/4	1230/12	1300/12	1350/12	Green Body
Density (g/cm^3^)	3.1	3.3	3.5	3.7	3.8	3.9	4.0	3.0
Relative Density	0.66	0.70	0.75	0.78	0.81	0.84	0.86	0.64
Total Porosity	0.34	0.30	0.25	0.22	0.19	0.16	0.14	0.36

**Table 4:** Dielectric permittivity, at different frequencies, for samples sintered at different conditions.

Frequency	Sample						
	1150/4	1200/4	1250/4	1300/4	1230/12	1300/12	1350/12
10^5^ Hz	36.0	39.0	39.6	41.8	44.9	48.3	49.5
10^6^ Hz	33.8	36.4	37.4	38.3	41.6	45.0	46.4
10^7^ Hz	31.8	34.5	36.44	37.2	39.2	42.3	43.8
1.29 GHz	-	-	-	-	-	-	59.9
2 GHz	-	-	-	-	-	-	58.2
